# Prehospital triage of patients with suspected acute stroke (PRESTO-2): protocol for a controlled before-and-after intervention trial

**DOI:** 10.1093/esj/aakag106

**Published:** 2026-09-07

**Authors:** Ruben M van de Wijdeven, Naomi S de Ruijter, Femke C C Kremers, Esmee Venema, Patrick J van der Geest, Johannes C M IJzermans, Merel Willeboer, Kirsten R I S Dorresteijn, Annemarie D Wijnhoud, Bas van der Veen, Jonathan M Coutinho, Diederik W J Dippel, Henk Kerkhoff, Bob Roozenbeek

**Affiliations:** Department of Neurology, Erasmus University Medical Center, Rotterdam, the Netherlands; Department of Neurology, Erasmus University Medical Center, Rotterdam, the Netherlands; Department of Neurology, Erasmus University Medical Center, Rotterdam, the Netherlands; Department of Neurology, Erasmus University Medical Center, Rotterdam, the Netherlands; Emergency Department, Erasmus University Medical Center, Rotterdam, the Netherlands; Department of Public Health, Erasmus University Medical Center, Rotterdam, the Netherlands; Ambulance Rotterdam-Rijnmond, Barendrecht, the Netherlands; Ambulance Service Zuid-Holland Zuid, Papendrecht, the Netherlands; Ambulance Service Zuid-Holland Zuid, Papendrecht, the Netherlands; Department of Neurology, Franciscus Gasthuis & Vlietland, Rotterdam, the Netherlands; Department of Neurology, IJsselland Hospital, Capelle aan den IJssel, the Netherlands; Department of Neurology, Noordwest Ziekenhuisgroep, Alkmaar, the Netherlands; Department of Neurology, Amsterdam UMC, Amsterdam, the Netherlands; Department of Neurology, Erasmus University Medical Center, Rotterdam, the Netherlands; Department of Neurology, Albert Schweitzer Hospital, Dordrecht, the Netherlands; Department of Neurology, Erasmus University Medical Center, Rotterdam, the Netherlands

**Keywords:** acute ischaemic stroke, digital health tools, EVT, healthcare organisation, prehospital triage

## Abstract

**Background:**

Prehospital triage of suspected stroke patients aims to reduce onset-to-groin time by eliminating the need for interhospital transfers for patients with ischaemic stroke caused by an LVO eligible for EVT, thereby improving long-term functional outcome. We aim to evaluate the implementation of a novel decision support tool, the Stroke Triage App (STA), which provides personalised triage advice by integrating stroke severity, onset time, GPS-based driving times, hospital-specific workflow metrics and time-dependent treatment effects.

**Study design:**

PRESTO-2 is a multicentre controlled before-and-after intervention trial. The study aims to evaluate the outcomes before and after implementation of prehospital triage with the STA in 1 acute-care region in the south-west of the Netherlands, compared with a control region. The STA is a smartphone-based application that uses an integrated decision model to estimate the probability of good functional outcome after 3 months (mRS score 0–2) under 2 strategies: transportation to the nearest primary stroke centre or transportation to the more distant intervention centre. Paramedics will use the STA for suspected stroke patients with a positive FAST test of 1 point or more.

**Study endpoints:**

The primary outcome is onset-to-groin time. Effects will be estimated using difference-in-differences linear regression analysis. Secondary outcomes include the onset-to-needle time, 3-month functional outcome, proportion of patients treated with IVT and EVT within guideline-recommended time windows, overtriage and undertriage, ambulance utilisation, compliance and adherence to triage advice.

**Summary:**

The PRESTO-2 study uses a pragmatic and efficient design for implementation studies, and is expected to provide insights into the optimisation of prehospital stroke care pathways and the challenges associated with integration of novel digital health technologies in existing clinical practice. These findings may inform scientists and clinicians to more efficiently design studies and organise stroke care pathways.

## Graphical Abstract

**Graphical Abstract:**
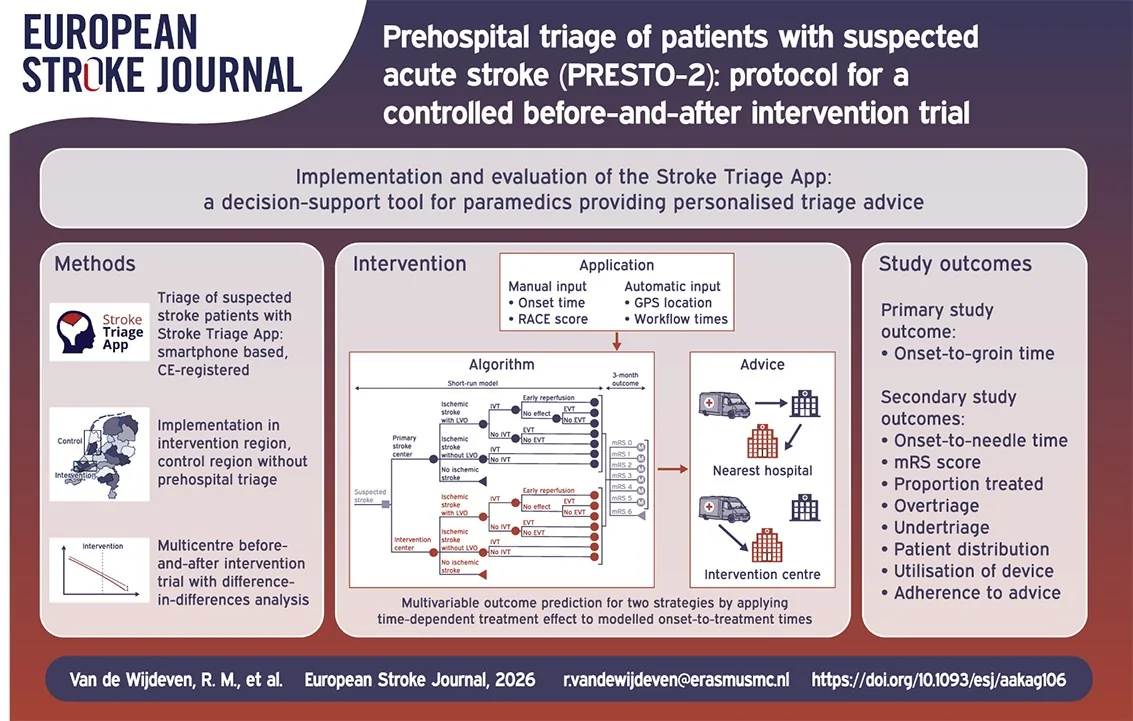

## Introduction

The effectiveness of both available acute reperfusion therapies for ischaemic stroke, intravenous thrombolysis (IVT) and endovascular thrombectomy (EVT), decreases as time elapses.^1–5^ IVT is available in most primary stroke centres (PSCs) and has a relatively broad indication, while EVT is only available in specialised intervention centres and is restricted to patients with LVO.^6–9^ Approximately one-quarter of patients with acute ischaemic stroke have a proximal intracranial LVO.^10^ The effect of IVT in this group of patients is small, but EVT is highly efficacious.^7^^,^^8^^,^^10^^,^^11^

Interhospital transfers are required for patients with an LVO stroke when they have first been transported to the nearest PSC, causing delay to initiation of EVT.^12^^,^^13^ Prehospital triage aims to eliminate these interhospital transfers by identifying these patients in the ambulance, allowing immediate transportation to an intervention centre. Various tools for detecting or estimating the likelihood of LVO have been developed to facilitate prehospital triage. Among these tools, prehospital stroke scales are the most widely used and investigated.^10^^,^^14^ When the total score of the prehospital stroke scale for a patient is above a defined cut-point, the patient is directly transported to the intervention centre.^15^^,^^16^ Prehospital stroke scales are implemented in several ambulance regions in the Netherlands and beyond to facilitate prehospital triage decision making, although a benefit of implementation of these scales on long-term functional outcome has not been unequivocally established.^17^ These scales do not account for essential regional characteristics, such as driving times to hospitals, workflow times within hospitals and transfer times between hospitals, while previous research showed that these are also important factors in determining the optimal transportation strategy besides stroke severity.^13^ These factors are combined with one of the best performing prehospital LVO detection scales, the Rapid Arterial oCclusion Evaluation (RACE) scale, into a decision support tool, the Stroke Triage App.^10^^,^^13^^,^^14^^,^^18^ The Stroke Triage App consists of an algorithm combining these factors in a multivariable decision model to aid ambulance staff in determining the best transport strategy for each individual patient.^19^^,^^20^

### Objectives

The primary objective of the PRESTO-2 study is to evaluate whether regional implementation of prehospital triage with the Stroke Triage App, compared with standard prehospital care without triage optimisation (ie, a drip-and-ship strategy), is associated with changes in onset-to-groin time in patients with ischaemic stroke due to LVO undergoing EVT, and with changes in onset-to-needle time in patients with ischaemic stroke without LVO. The secondary objective is to evaluate the implementation of the Stroke Triage App in the intervention region, in terms of logistics (over- and undertriage proportions and patient distribution), patient outcomes (proportion of patients treated with IVT and EVT) and user metrics (app usage, number of users and compliance among paramedics).

## Methods

### Study design

The prehospital triage of patients with a suspected acute stroke (PRESTO-2) study is a prospective multicentre non-randomised controlled before-and-after intervention trial. The primary and secondary objectives require different analytical approaches and are therefore described separately below. For the primary objective, we will perform an implementation study using a controlled before-and-after intervention design with difference-in-differences analysis. We will use data from an intervention region and a control region. For the secondary objective, we will conduct a prospective observational cohort study.

### The Stroke Triage App

**Figure 1:**
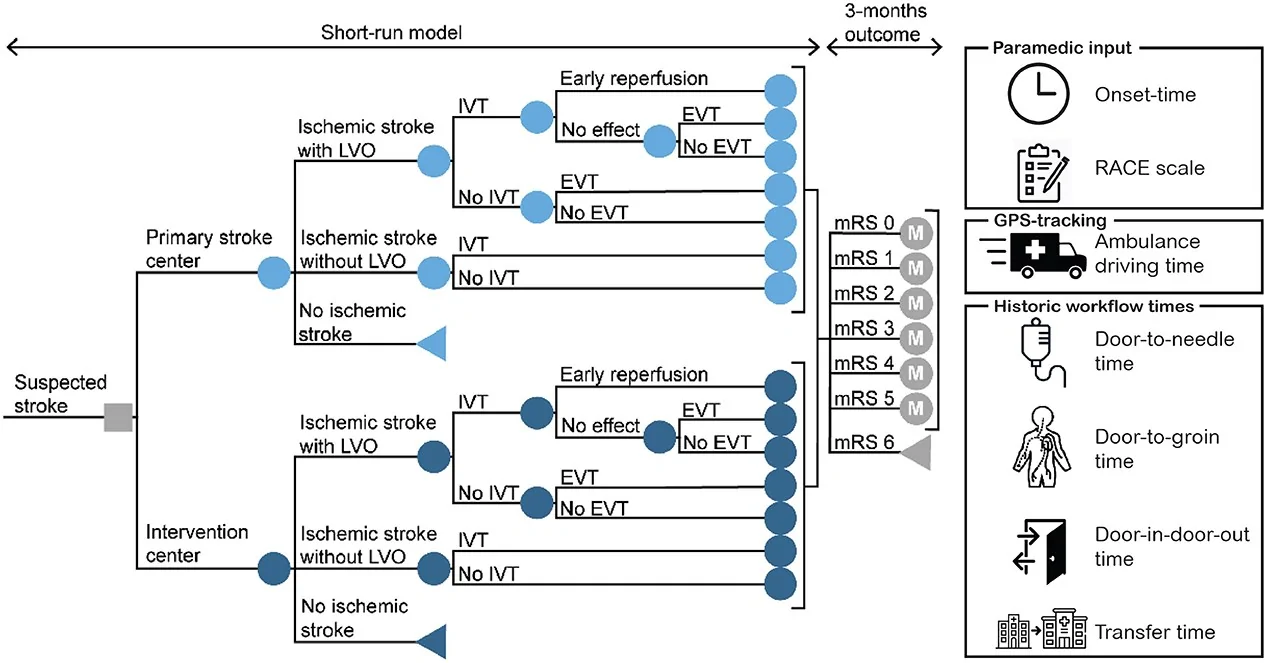
Visual representation of the decision model forming the basis of the algorithm behind the Stroke Triage App.

The decision model underlying the Stroke Triage App was derived from prior modelling studies and incorporates stroke severity assessed with the RACE scale, estimated symptom onset or last known well time, GPS-based transport times, interhospital transfer times and hospital-specific workflow metrics. For each patient, the app estimates the probability of good functional outcome (90-day mRS 0–2) under 2 transport strategies: transport to the nearest stroke centre (primary stroke centre or intervention centre) or direct transport to an intervention centre, bypassing closer primary stroke centres if appropriate.^19^^,^^21^ The app recommends the strategy with the highest probability of achieving an mRS score of 0–2 (Figure 1). The Stroke Triage App has been developed and is CE-certified under the Medical Device Regulation (MDR) class I software as medical device (EUDAMED UDI-DI: 08720892217905). Documentation regarding the MDR risk classification can be found in the Supplementary Materials.

### Study population

For the primary objective, we will include all adult patients (≥18 years) with a clinical hospital-confirmed diagnosis of ischaemic stroke who presented to the Emergency Department within 6 h from symptom onset in the 24 months before implementation (January 2022–January 2024) and 24 months after implementation (January 2024–January 2026) of the Stroke Triage App in both the intervention region and the control region. The intervention region consists of approximately 1.8 million inhabitants and 2 ambulance regions (Rotterdam-Rijnmond and Zuid-Holland Zuid), containing 2 intervention centres and 7 referring PSCs. The control region consists of approximately 2.8 million inhabitants and 4 ambulance regions (Zaanstreek-Waterland, Kennemerland, Amsterdam-Amstelland and Noord-Holland-Noord), containing 2 intervention centres and 9 referring PSCs, of which one closed in early 2023. No form of individualised triage was implemented in the control region during the study period, with patients being transported to the nearest IVT-capable stroke centre in accordance with national guidelines. The 2 regions are similar concerning degree of urbanisation and distances between hospitals (Tables 1 and 2).

**Table 1:** Description of the intervention region.

Hospital	Distance from intervention centre (km)	Estimated driving time (min) ^a^
**Erasmus MC**		
**Ikazia Ziekenhuis**	4.5	10
**Franciscus Gasthuis & Vlietland, location Gasthuis**	5.4	14
**Franciscus Gasthuis & Vlietland, location Vlietland**	7.5	14
**Maasstad Ziekenhuis**	8.9	20
**IJsselland Ziekenhuis**	10.4	22
**Van Weel Bethesda Ziekenhuis**	55.0	49
**Albert Schweitzer Ziekenhuis**		
**Beatrixziekenhuis**	25.4	23

^a^Estimated with ArcGIS Online Software version 10.6.1.

**Table 2:** Description of the control region.

Hospital	Distance from intervention centre (km)	Estimated driving time (min) ^a^
**Amsterdam UMC, location AMC**		
**Amsterdam UMC, location VUmc** ^**b**^	10.0	10
**OLVG West**	15.3	14
**Spaarne Gasthuis**	27.9	21
**Flevoziekenhuis**	29.3	24
**Rode Kruis Ziekenhuis**	35.9	26
**Dijklander Ziekenhuis, location Purmerend**	38.3	28
**Dijklander Ziekenhuis, location Hoorn**	56.8	37
**Noordwest Ziekenhuisgroep, location Alkmaar**		
**Noordwest Ziekenhuisgroep, location Den Helder**	43.1	43

^a^Estimated with ArcGIS Online Software version 10.6.1.

^b^Closed for acute stroke care in the spring of 2023.

For the secondary objective, we will include all consecutive adult patients (≥18 years) with a suspected stroke (based on a positive FAST-test [≥1 point, except for isolated dysarthria]) with onset of symptoms within the last 24 h for whom the Stroke Triage App was utilised in the intervention region during the study period.

### Data collection

Two separate datasets will be collected for the analysis of the 2 objectives. For the primary objective, data will be collected from the national stroke care quality registry (the Dutch Acute Stroke Audit [DASA] of the Dutch Institute of Clinical Auditing [DICA]). All Dutch hospitals report data regarding patient characteristics, process and outcome indicators to this registry annually. These registry data are used to prevent bias and disparities in the before-and-after intervention population cohorts in the intervention region and to control for time-dependent improvement in treatment times. To minimise the risk of double inclusions, patients receiving IVT will be included only if they were not transferred between hospitals. Patients undergoing EVT will be included solely from intervention centres. We will collect data including clinical characteristics (age, sex, type of stroke onset, date and time of stroke onset, NIHSS score and diagnosis), workflow and treatment indicators (date and time of presentation at Emergency Department, given treatment, door-to-needle time in case of IVT, door-to-groin time in case of EVT) and outcome indicators (mRS score after 3 months).

For the secondary objective, we will collect data gathered in the ambulance that is entered into the Stroke Triage App, consisting of the ambulance ride number, GPS location, time of symptom onset or last known well and individual RACE scale items. We will also collect data which are automatically generated by the Stroke Triage App, consisting of the onset-to-departure time, probability of LVO based on the RACE scale sum score and driving times to various hospitals, as well as workflow metrics consisting of login time and total duration of triage. The ambulance ride number is used to identify patients from the EMS databases, in order to collect data regarding demographics, vital functions, general examination, transportation times and complications (ie, vomiting, aspiration and neurological deterioration) occurring during transportation. After transportation to the hospital, suspected stroke patients will undergo standard diagnostic work-up at the discretion of the attending physician. This work-up may consist of laboratory testing, non-contrast enhanced CT scan, CTA, CTP and/or digital subtraction angiography. No additional testing is performed within the context of this study. We will collect data regarding medical history, physical and neurological examination, diagnostic tests, diagnosis and administered treatment, including specific workflow times when applicable, from the hospital electronic health records.

### Outcome measures

#### Primary objective

The primary outcome measure for the primary objective will be the symptom onset or last known well time to groin puncture time (start of EVT) in patients with an ischaemic stroke with LVO who presented in the emergency department within 6 h from stroke onset. Secondary outcome measures for the primary objective will be the symptom onset to needle time (start of IVT) in patients who are treated with IVT, the percentage of patients with ischaemic stroke treated with IVT within the conventional guideline-recommended time window of 4.5 h after symptom onset or last seen well, the percentage of patients with ischaemic stroke treated with EVT within the conventional guideline-recommended time window of 6 h after symptom-onset or last seen well and the mRS score after 3 months.

#### Secondary objective

For the secondary objective, outcome measures will be the distribution of final diagnoses (ischaemic stroke with LVO, ischaemic stroke without LVO, TIA, intracerebral haemorrhage and stroke mimics) in PSC and intervention centres, the number and percentage of ischaemic stroke patients without EVT-eligible occlusion taken to an intervention centre after bypassing a PSC (overtriage), the number and percentage of ischaemic stroke patients with an EVT-eligible occlusion who were not bypassed to an intervention centre (undertriage), the number of triages per month, number of users per month, number of uses per paramedic per month, compliance, adherence to the Stroke Triage App advice, total number of required acute ambulance transportations (prior to reperfusion therapy) and total number of required subacute ambulance transportations (transfers after reperfusion therapy or in absence of an indication thereof).

### Sample size calculation

Under the initial assumption that the Stroke Triage App would be used for all eligible cases and within the context of an implementation study running for 12 months, a power calculation rather than a sample size calculation was conducted. A mean (±SD) onset-to-groin time of 224.5 (±94.1) min was extracted from the Dutch MR CLEAN registry. Based on modelled data, where the Stroke Triage App algorithm has been applied to a prospectively collected cohort of prehospital suspected stroke patients, the mean effect of implementation of triage with the Stroke Triage App algorithm on the primary outcome measure (onset-to-groin time in patients undergoing EVT for ischaemic stroke with LVO) in the intervention region is estimated to be −31 min.^21^ We assumed a low intra-cluster correlation coefficient (ICC) of 0.01, as the primary outcome, onset-to-groin time, is substantially influenced by onset-to-alarm time. This interval is highly variable and largely determined by patient-specific and situational factors, such as stroke severity, aphasia, the presence of witnesses and time of symptom onset, which are largely independent of cluster membership. We also expected limited between-cluster variability. Public stroke-awareness campaigns are implemented nationally, acute stroke care is standardised through national guidelines and the study regions are comparable in terms of urbanisation. Together, the high within-cluster variability and limited between-cluster variability support the assumption of a low ICC in this study.

Based on previous stroke registry analyses, 96 EVTs annually in the intervention region would be required to reach a power of 80%. It was estimated that approximately 400 EVTs take place annually in the intervention region, which corresponds to a power of > 99% at a predefined alpha of 0.05.^22^ A sample size analysis for the secondary objective was not applicable.

### Data analysis plan

The statistical analysis will be performed after inclusion of the last patient, after the data have been checked, errors have been corrected and the database has been locked. Missing data will be handled via simple imputation (if < 5% missing) or multiple imputation by means of a multiple regression model (if ≥ 5% missing). We will estimate the effect of the implementation of the Stroke Triage App on the primary and secondary outcomes of the primary objective using difference-in-differences linear regression analysis. This analysis compares the differences before and after implementation in the implementation region with the differences between the same periods in the control region where no implementation takes place. This method adjusts for unobserved confounders and (other) time-related differences in acute stroke care. For the secondary objective, we will report the observed outcomes in the study cohort. We will then conduct counterfactual simulation assuming transport to the nearest hospital, in accordance with the ambulance protocol that was in place prior to the implementation of the Stroke Triage App (“drip-and-ship” model). We will compare the observed outcomes with the simulated outcomes in order to be able to make a statement about the effects of the implementation of the Stroke Triage App on these outcomes. Closure of the database and analysis of the data is planned after submission of the protocol for publication. A detailed Statistical Analysis Plan can be found in the Supplementary Materials.

### Patient and public partnership

Patients and the public were not involved in the design of this study. The Erasmus MC Stroke Center Patient Panel was involved in the design of patient information forms and was consulted regarding the opt-out procedure. A user council consisting of paramedics was established and consulted during various phases of the study, including development, implementation and evaluation of the application. Furthermore, the study protocol, patient information forms, progress of the study and specific information for patients, researchers and healthcare professionals are publicly available on the project website (www.stroketriageapp.com).

### Duration and current status of the study

The study started on 22nd January 2024, when the Stroke Triage App went live and was incorporated into the regional protocols for ambulance care. Due to lower-than-expected compliance and usage of the Stroke Triage App, the study protocol was amended to facilitate an interim analysis allowing for re-estimation of the sample size. During this interim analysis at 9 months after implementation, the usage rate of the Stroke Triage App was evaluated. It was determined that the Stroke Triage App was used for triage in 33% of patients eligible for EVT. Based on the revised effect size of the intervention, a new sample size calculation was conducted. Assuming a reduced effect size of 33%, we estimated that the Stroke Triage App would need to be used for triage of 274 patients eligible for EVT in the national DASA registry. Therefore, the study group decided that the study duration could be prolonged by a maximum of 12 months. Initially, enrolment was planned to finish on 22nd January 2025. This deadline was extended to 31st December 2025 to meet the requirement of the new sample size calculation.

### Ethical aspects and informed consent

The study will be conducted according to the principles of the Declaration of Helsinki (October 2013), Gedragscode Gezondheidsonderzoek 2022, the Dutch Medical Treatment Contracts Act (WGBO) and the General Data Protection Regulation (GDPR). The Stroke Triage App is an MDR risk class I CE-marked software as a medical device used within the scope of its intended purpose. This study is part of post-market clinical follow-up and does not comprise additional invasive or burdensome procedures compared to standard of care. As such, it falls outside the scope of chapter VI of the MDR (EU no 2017/745). The Institutional Review Board of the Erasmus MC has reviewed the study protocol and confirmed that the Dutch Medical Research Involving Human Subjects Act (WMO) is not applicable.

For the primary objective, non-personally identifiable data collected as part of a national quality registry (Dutch Acute Stroke Audit) will be used, in which all Dutch hospitals participate. These data are collected with an exemption from patient consent, as is common practice in clinical audits. Hospitals are free to determine who carries out the data registration (for example, [research] nurses, data managers or neurologists), but the final responsibility lies with the neurologist.

For the secondary objective, paramedics include patients with suspected stroke. Many patients with a stroke have a language impairment, anosognosia or other cognitive symptoms that complicate an informed consent procedure. A representative of the patient is often not immediately present in the prehospital setting. Moreover, a proper informed consent procedure takes time, which is not available in the ambulance. Taking informed consent afterwards (“deferred consent”) during hospitalisation is burdensome for both the patient and family and would have to be conducted by the attending physician within the context of the WGBO. Because the patients spread in various directions after the initial ambulance ride, a disproportionate number of healthcare providers or physicians from a multitude of specialties (neurologists, emergency physicians, internists, geriatricians, cardiologists and general practitioners) must be involved in order for the deferred consent procedure to take place. Because consent cannot reasonably be requested given the nature and purpose of the research, an appeal is made to the exception of Section 7:458(1)(b) of the Dutch Civil Code, as stated in Chapter 5, Section 5.4 of the 2022 Code of Conduct for Health Research. The magnitude of the effort that must be made by a large number of healthcare providers to obtain consent from the participating patients is disproportionate in relation to the relatively limited sensitivity of the collected personal data and the associated limited infringement of the personal living atmosphere. Moreover, the risks of participating in the study are minimal and using a deferred consent procedure could reasonably be presumed to lead to a drop-out of selected groups of patients due to geographical and/or clinical characteristics. This could jeopardise the validity and the reliability of the results and may lead to erroneous conclusions, making it unethical to conduct the study altogether.

Because of the aforementioned reasons, an opt-out procedure will be used. The attending paramedic will provide a patient information form in which the patient is informed about the study. This form also states that data has been collected in the ambulance and that routinely collected data from the hospital will be requested for further analysis. Patients or their next of kin are given the opportunity to object to the use of these data for the study. When a patient or next of kin objects, all data will be destroyed and this patient will no longer be included in the study.

### Dissemination plan

The study results will be disseminated to the broader public through publication in international peer-reviewed journals and by presentations at international conferences. Representatives of the participating hospitals and ambulance services will be invited as co-authors as stipulated in the data transfer agreements and asked to critically review the manuscript, in accordance with the recommendations of the International Committee of Medical Journal Editors. We plan to disseminate the results of planned secondary analyses in multiple separate manuscripts. The opt-out procedure does not provide for sharing of data with other research groups in the future, but collaborations will be possible by reaching out to the primary investigator.

## Discussion

In recent years, a variety of tools have been developed to aid paramedics in prehospital transportation decision-making for patients with acute ischaemic stroke. These tools were initially developed to rapidly identify patients with EVT-eligible stroke in order to allow direct transportation to an intervention centre, whilst ensuring that patients with non-EVT-eligible stroke are not subjected to unnecessary delays in care.^23^ Despite significant advances in prehospital diagnostic tools, optimisation of prehospital stroke care pathways remains challenging and studies investigating the implementation of prehospital stroke scales have reported heterogeneous results.^17^^,^^24^ This may in part be explained because other factors, such as stroke severity, geographical factors, driving times and transfer times and hospital-specific workflow times, were unaccounted for.^19^^,^^25^ Many of these tools fail to adequately incorporate these critical region-specific factors, limiting their applicability across existing heterogeneous stroke-care systems.^26^

Within this context, this study has several notable strengths. The Stroke Triage App contains a decision model incorporating the most important aforementioned variables to calculate the probability of a good functional outcome for 2 transportation strategies for each individual patient. This personalised approach to prehospital triage allows for the Stroke Triage App to more easily be implemented in other regions with different regional characteristics. Prior to implementation of the Stroke Triage App, the effectiveness of the triage algorithm was established through modelling in a real-world cohort of suspected stroke patients in the region, supporting implementation of the application as a CE-compliant medical device.^21^ To evaluate the effectiveness of implementation of the Stroke Triage App, we considered utilising a randomised controlled trial design, but this approach would come with significant drawbacks. First, patient inclusion and randomisation to different transportation strategies would be a time-consuming process, adversely affecting the study population with treatment delays. Second, individual patient randomisation would likely have resulted in contamination of the control arm. After training in the use of the Stroke Triage App, paramedics may consciously or subconsciously incorporate the app’s triage logic into decision-making for patients allocated to standard care. This would diminish the contrast between intervention and control conditions and compromise the ability of the study to estimate the true effect of implementation. Third, the study aimed to evaluate the effectiveness of actual implementation of prehospital triage with the Stroke Triage App, including factors such as suboptimal paramedic compliance and non-adherence to the advice due to unforeseen circumstances. Finally, obtaining informed consent prior to or deferred consent after inclusion posed substantial logistical challenges, as previously described in the Methods section. Given that the Stroke Triage App was implemented as part of routine clinical care, we chose to utilise a pragmatic, non-randomised controlled before-and-after intervention design instead. This quasi-experimental design enables comparison with a control group, while the difference-in-differences analysis also adjusts for temporal trends in the improvement of treatment times for acute ischaemic stroke. One challenge in the study design was identifying a comparable control region without prehospital triage. Although distances between PSCs and intervention centres differed slightly between regions, these differences were stable over time and are therefore unlikely to bias the estimated intervention effect. As such, we expect the between-region differences before and after implementation to primarily reflect underlying temporal trends. Another challenge of this study was selecting an appropriate outcome measure. Onset-to-groin time in patients undergoing EVT reflects the potential benefits of the intervention, whereas onset-to-needle time in patients receiving IVT reflects its potential adverse consequences. A composite outcome was considered, but this was not feasible due to the expected high proportion of missing 3-month mRS scores in the DASA registry. Moreover, combining these time-based outcomes was methodologically challenging, as minutes gained in onset-to-groin time are not directly comparable to minutes lost in onset-to-needle time. To account for patients who may have forgone treatment as a result of triage decisions, we additionally report the proportion of patients receiving EVT and IVT.

In summary, the PRESTO-2 study is distinctive in its evaluation of real-world implementation of a novel prehospital triage tool, the Stroke Triage App, in patients with suspected stroke. The tool incorporates a decision model that integrates various essential variables such as stroke severity, time since stroke onset, driving times to various hospitals, transfer times and hospital-specific workflow times, in order to generate a transport advice for paramedics. This personalised approach and adaptable framework facilitates implementation across regions with diverse healthcare infrastructures. This study is expected to provide valuable insights into the optimisation of organisation of prehospital stroke care pathways. Moreover, it contributes to a broader understanding of the challenges and opportunities associated with implementing digital health technologies in routine clinical practice. Lastly, the innovative design of the PRESTO-2 study demonstrates that complex implementation studies can be conducted in an efficient and pragmatic manner. In the context of declining availability of research funding and increasing pressure on healthcare accessibility, such innovations may play a pivotal role in sustaining efficient and equitable care delivery.

## Supplementary Material

Supplementary_material_aakag106

## Data Availability

Individual patient-level data will not be transferred to external researchers, as explicit patient consent for external data sharing was not obtained. However, proposals for secondary analyses may be submitted to the PRESTO-2 investigators. Following approval of a research proposal and subject to applicable data protection and governance requirements, approved analyses may be facilitated within a secure data environment without transfer of individual-level data to external researchers. Study materials, including patient information leaflets and paramedic training materials, are available from the corresponding author upon reasonable request.
