# Supplementary material for: Prehospital triage of patients with suspected acute stroke (PRESTO-2): protocol for a controlled before-and-after intervention trial
Source: Eur Stroke J. 2026 Sep 7;11(9):aakag106. doi: 10.1093/esj/aakag106 (PMC13547856; doi:10.1093/esj/aakag106)
Supplement: Supplementary file 1 [file supplementary_material_aakag106.zip › Supplements_-_Protocol_paper_PRESTO-2_-_20260727.pdf]

SUPPLEMENTAL MATERIAL

Document 1. Stroke Triage App – Intended Purpose and Classification

Document 2. Statistical analysis plan

Stroke Triage App
Intended Purpose and Classification

Version: *3.0* | ***Initially created:*** *2 May 2023* | ***Last updated:*** *16 July 2026*

The scope of this document is to define the intended purpose, software qualification and classification of the Stroke Triage App.

Intended purpose

Intended use

The Stroke Triage App advises paramedics on the type of hospital a patient with a suspected stroke should be transported to. The Stroke Triage App's advice is based on a comparison of patient prognosis for two different strategies: transport to nearest primary stroke center or intervention center. The prognosis is derived from the duration of symptoms, the severity of neurological symptoms (expressed in the RACE scale score), geographical location, current driving times and working speed within the hospitals, which are then combined to model the onset-to-treatment for both strategies and weighed with the time-dependent treatment effect. The diagnostic and therapeutic decision is then drawn up in the hospital by the attending physicians, regardless of the transportation strategy chosen. The aim of the Stroke Triage App is to provide paramedics with a substantiated transportation advice, in order to minimize interhospital transfers, thereby decreasing the average time to treatment for patients and increasing the probability of good outcome.

Additionally, the calculated RACE score is used as an input for modelling patient prognosis and is communicated to the destination hospital as a prenotification, to allow allocation and preparation of the required resources before clinical assessment begins. The RACE score is not intended to be used for diagnosis, treatment decisions, or assessing eligibility for any treatment (e.g. endovascular therapy or intravenous thrombolysis). All diagnostic and therapeutic decisions are made by physicians in the hospital, regardless of the transportation strategy chosen by the Stroke Triage App.

Medical indications and contra-indications

The Stroke Triage App is indicated for use in patients aged 18 years and older with suspected cerebrovascular accident (CVA), based on a positive FAST test (excluding isolated dysarthria), where symptoms have existed for less than 24 hours.

Contra-indications: no contra-indications are known for the use of this medical device. The Stroke Triage App supports logistical triage and does not replace professional clinical judgment. The ambulance professional should always include the overall clinical condition of the patient in decision-making. In situations where the triage outcome is deemed inappropriate, clinical judgment should take precedence.

Target patient population

Patient group for whom the use of Stroke Triage is intended:

- Adult patients (≥ 18 years)
- Patients with:
    - acute neurological deficits consistent with CVA (excl. isolated dysarthria)
    - a positive FAST test
    - symptoms existing for less than 24 hours
- Patients in the prehospital setting, assessed by an ambulance nurse

MDR Qualification

Stroke Triage qualifies as a **medical device** based on Article 2(1) of Regulation (EU) 2017/745 (MDR) and the MDSW qualification and classification decision tree as described in MDCG 2019-11, based on the following rationale:

Does your software have a medical or health purpose (art. 2 sub 1 MDR) for the benefit of individual patients?

Yes, the Stroke Triage App is software that provides information intended to be used for human beings for the following medical purpose: advise on the best transportation strategy based on prognosis of suspected stroke patients.

Is the software performing an action on data different from storage, archival, communication, simple search, or lossless compression?

Yes, the Stroke Triage App performs an action on data different from storage, archival, communication, simple search, or lossless compression, for the benefit of individual patients; the application advises paramedics to transport a patient with a suspected stroke to the nearest primary stroke center or intervention center, based on prognosis. Patient prognosis is estimated from duration of symptoms, the severity of neurological symptoms (expressed in the RACE scale score), geographical location, current driving times and working speed within the

hospitals, which are then combined to model the onset-to-treatment and weighed with the time-dependent treatment effect. Additionally, the RACE score is used for prenotification to allocate the required resources at the hospital.

MDR Software Classification

Stroke Triage classifies as a **class I** medical device in accordance with Rule 11 of Annex VIII to Regulation (EU) 2017/745 (MDR) and the MDSW classification guidance in MDCG 2019-11, based on the following rationale:

Does your software provide information used to make diagnostic or therapeutic decisions?

No, the Stroke Triage App is not intended to provide information which is used to take decisions with diagnosis or therapeutic purposes. The provided information is used for transportation strategy only, which makes the Stroke Triage App "all other software classified as class I". The prediction model in the Stroke Triage App compares the prognosis (probability of good functional outcome after 3 months) of two transportation strategies and returns the one that has the highest probability of good outcome. The Stroke Triage App specifically does not give advice regarding diagnosis or therapy. Upon arrival at the Emergency Department, all patients will receive diagnostic work-up and treatment as deemed appropriate by the attending physician, regardless of the Stroke Triage App's advice. The paramedic will be instructed to communicate the RACE score with the hospital. This RACE score is not used to make diagnostic or therapeutic decisions in the hospital by the physician. The purpose of communicating the RACE score is to serve as a prenotification to the destination hospital. This prenotification allows the hospital to allocate and prepare the required resources (personnel, CT-room, ED-room, angiosuite) for the work-up of the suspected stroke patient.

Does your software monitor physiological processes (heart rate, respirations, menstrual cycle, etc.)? (Rule 11, Annex VIII MDR)

No, the Stroke Triage App does not monitor physiological processes.

The MDCG 2019-11 classification guidance table for Rule 11 of Annex VIII MDR has been consulted; however, its higher classifications (Class IIa and above) do not apply because the software does not provide information used to make diagnostic or therapeutic decisions. The Stroke Triage App therefore falls under "all other software", which is classified as Class I.

ORTEC B.V.
https://stroketriageapp.com/

Statistical analysis plan

Prehospital triage of patients with a suspected acute stroke (PRESTO-2)

**Project leaders**	B. Roozenbeek, MD, PhD<br>Erasmus MC<br>Department of Neurology<br>Dr. Molewaterplein 40, 3015GD,<br>Rotterdam, NL Email:<br>b.roozenbeek@erasmusmc.nl<br><br>H. Kerkhoff, MD, PhD<br>Albert Schweitzer hospital<br>Department of Neurology<br>Albert Schweitzerplaats 25, 3318AT,<br>Dordrecht, NL Email: h.kerkhoff@asz.nl
**Coordinating investigators**	F.C.C. Kremers, MD<br>N.S. de Ruijter, MD<br>Erasmus MC<br>Department of Neurology<br>Dr. Molewaterplein 40, 3015GD,<br>Rotterdam, the Netherlands Email:<br>f.kremers@erasmusmc.nl<br>n.deruijter@erasmusmc.nl
**Sponsor**	Erasmus MC University Medical Center<br>Rotterdam Dr. Molewaterplein 40<br>3015 CD Rotterdam, the Netherlands
**Subsidizing party**	ZonMw, Dutch Heart Foundation
**Author**	F.C.C. Kremers, MD and N.S. de<br>Ruijter, MD

Table of Contents

List of abbreviations and relevant definitions 4
1. Introduction and rationale 5
2. Objective(s) 5
3. Study type 6
4. Study population 6
5. Methods 6
6. Incidental findings 6
7. Burden and risks 7
8. Recruitment and consent 7
9. Statistical analysis plan 8

List of abbreviations and relevant definitions

**CE**	Conformité Européene
**CTA**	Clinical Trial Agreement
**DMP**	Data Management Plan
**DPIA**	Data Protection Impact Assessment
**DTA**	Data Transfer Agreement
**EVT**	Endovascular thrombectomy
**Exception consent**	Form Care for data Template, in Dutch: Formulier uitzondering toestemming
**FAST**	Face Arm Speech Time
**GCP**	Good Clinical Practice
**GCS**	Glasgow Coma Scale
**GDPR**	General Data Protection Regulation in Dutch: Algemene Verordening Gegevensbescherming
**GPS**	Global Positioning System
**IC**	Informed Consent
**IVT**	Intravenous thrombolytics
**IFU**	Instruction For Use
**LVO**	Large vessel occlusion
**mRS**	modified Rankin Scale
**MTA**	Material Transfer Agreement
**NIHSS**	National Institutes of Health Stroke Scale
**NWTC**	Non-WMO Review Committee; in Dutch: Niet WMO Toetsingscommissie
**PSC**	Primary stroke center
**STA**	**Stroke Triage App**
**RACE**	Rapid Arterial oCclusion Evaluation
**ROAZ**	Regionaal Overleg Acute Zorg

1. Introduction and rationale

In current practice, patients with a suspected stroke are transported by ambulance to the nearest stroke center capable of administering intravenous thrombolytics (IVT). Patients presenting with a large vessel occlusion (LVO) at presentation in a primary (=non-intervention) stroke center are transferred to an intervention center for endovascular treatment (EVT). Such transfers delay the start of EVT and adversely affect functional recovery. An alternative strategy is to transport all patients with suspected stroke directly to an intervention center. However, this approach may delay treatment with IVT. Triage of patients eligible for EVT in the prehospital setting may reduce the need for interhospital transfers, thereby expediting the time to EVT and improving long-term functional outcomes. The Stroke Triage App (STA) has been developed to provide a substantiated transportation advice to paramedics. The STA provides personalized prehospital triage advice by integrating stroke severity, onset time, GPS-based driving times, hospital-specific workflow metrics, and time-dependent treatment effects. It compares two transportation strategies (transfer to the nearest primary stroke center and transfer to an intervention center) and identifies the strategy which yields the highest estimated probability of achieving a favorable long-term functional outcome.

2. Objective(s)

The overall objective of the PRESTO-2 study is to evaluate the implementation of the Stroke Triage App, a decision support tool designed for individualized prehospital triage of patients with suspected stroke. We defined the following objectives:

1. To assess whether implementation of prehospital triage with the Stroke Triage App reduces the onset-to-groin time in patients with ischemic stroke due to large vessel occlusion, and whether implementation of the Stroke Triage App impacts the onset-to-needle time in patients without a large vessel occlusion.

2. To evaluate implementation of the Stroke Triage App in the intervention region, regarding (1) logistic measures, such as percentage over- and undertriage and patient distribution, (2) patient measures, such as the percentage of IVT/EVT within 4.5 and 6 hours respectively, complications and the distribution of diagnoses such as

intracerebral hemorrhage and the corresponding triage advice, distribution of functional outcome measured with the modified Rankin Scale (mRS) and (3) user measures, such as the number of monthly users of the Stroke Triage App, and compliance amongst paramedics.

3. Study type

For the first specific objective, we will perform an implementation study using a before-and-after intervention design with difference-in-differences analysis. We will use data from an intervention region and a control region.

For the second specific objective, we will describe the prospective observational cohort of patients with a suspected stroke for which the STA is used.

4. Study population

1. All consecutive adult patients (≥18 years) with a clinical diagnosis of ischemic stroke in the 24 months prior and 24 months after implementation of the Stroke Triage App, in the intervention region and the control region.

2. All consecutive adult patients (≥18 years) with a suspected stroke with onset of symptoms within the last 24 hours for whom the Stroke Triage App was utilized by paramedics to facilitate prehospital triage in the 24 months after implementation of the Stroke Triage App in the intervention region.

6. Methods

Paramedics use the Stroke Triage App in the ambulance to obtain routing advice for the patient transport. The primary outcome in objective 1 is the reduction in onset-to-groin time, assessed by calculating the difference-in-differences estimator with a difference-in-differences linear regression model with data from the national Dutch Audit (DASA) registry. With this data, the intervention region will be compared with a control region to adjust for time-dependent improvements in doo- to-groin times. The intervention region consists of the ambulance regions Rotterdam-Rijnmond and Zuid-Holland Zuid. The control region is defined as the ambulance regions

Zaanstreek-Waterland, Kennemerland, Amsterdam-Amstelland and Noord-Holland-Noord. A more detailed description is found in the appendix I of the nWMO protocol.

Regarding objective 2, descriptive analysis will be performed for all suspected stroke patients for whom the Stroke Triage App is used. Prehospital data will be enriched with data from the ambulance call reports and hospital electronic files. Outcomes that will be investigated, are:

1. Logistic metrics
    - Patient distribution, percentage under- and overtriage, correctly bypassed patients
2. Patient metrics
    - Percentage of EVT, IVT, and percentage of treatments within conventional treatment windows (IVT: <4,5 hours, EVT: <6 hours), door- to-groin, door-to-needle times
    - Complications and the distribution of diagnoses such as intracerebral hemorrhage and the corresponding triage advice
    - Distribution of functional outcome measured with the modified Rankin Scale (mRS)
3. User metrics
    - Monthly users of the Stroke Triage App, number of monthly triages, user compliance

7. Burden and risks

The burden on participants is minimal. The study procedures are conducted in the prehospital setting and consist of a brief neurological examination (with two additional items compared to current clinical practice) and transport to a hospital. There is a small risk that standard treatment with intravenous thrombolysis will be minimally delayed, but it is expected that this is outweighed by the time gained to standard treatment with endovascular thrombectomy.

8. Recruitment and consent

Patients are recruited in the prehospital setting by paramedics. Due to the nature of the study, an appeal is made to the exception of Section 7:458(1)(b) of the Dutch Civil Code, as stated in Chapter 5 Section 5.4 of the 2022 Code of Conduct for Health Research, for an exemption for the need of informed consent in accordance with the code of conduct 2022.

9. Statistical analysis plan

A statistical plan for analysis is developed for both objectives.

9.1 Statistical analysis of objective 1: *To assess whether implementation of prehospital triage with the Stroke Triage App reduces the onset-to-groin time in patients with ischemic stroke due to large vessel occlusion, and whether implementation of the Stroke Triage App impacts the onset-to-needle time in patients without a large vessel occlusion.*

For the primary objective, data will be collected from the national stroke care quality registry (the Dutch Acute Stroke Audit (DASA) of the Dutch Institute of Clinical Auditing (DICA)). This dataset contains all patients with a diagnosis of ischaemic stroke from 2022-2025, from all hospitals in the intervention and control region, for the time period before and after implementation of the Stroke Triage App. The Stroke Triage App only considers a direct transfer/bypass to an intervention center when it is used within the time window of 6 hours after symptom onset. Therefore, for this analysis, we will only select patients who were presented at the emergency department within 6 hours after symptom onset. If symptom onset is unknown, patients will be excluded for analysis of the primary outcome.

Missing data
We will report proportions of missing values for all described variables in the baseline characteristics overview. For descriptive analyses, only the crude, non-imputed data will be presented. For regression analyses, missing data will be imputed, with the choice between single and multiple imputation determined by the proportion of missing values and the presumed underlying mechanism of missingness.

Primary effect analysis
The primary outcome measure in objective 1 is the (reduction in) onset-to-groin time. Therefore, only patients with LVO ischemic stroke who are treated with EVT will be selected from the dataset. To avoid duplicate inclusions, we will delete patient data from primary (= non-intervention) stroke centers collected in the DASA registry. Subsequently, for every patient, we will calculate the time-interval between symptom onset and groin puncture (start of EVT).

The primary effect parameter will be a difference in onset-to-groin time with its 95% confidence interval before and after implementation of the Stroke Triage App, compared to a control region, to account for time-dependent improvements. This will be estimated with a difference-in-differences linear regression model. Before analysis, we will check whether the parallel trends assumption is met in both regions by means of a graphical comparison between onset-to-groin times the two regions before implementation, to support the difference-in-differences analysis.

Adjusted for:

- Age
- Sex
- NIHSS

Secondary effect analysis
The secondary outcome

measure for the primary objective will be the change of onset-to-needle time in patients who are treated with IVT. For this purpose, we will select all patients who underwent IVT, excluding the patients who were referred from primary stroke centers (to avoid duplicate inclusions) collected in the DASA registry. Just as with the primary outcome measure, the effect on the secondary outcome measure will be estimated with a difference-in-differences linear regression model. Model adjustment in the linear differences-in-differences model will be similar for the secondary outcome.

9.2 Statistical analysis of objective 2: *To evaluate implementation of the Stroke Triage App in suspected stroke patients in the intervention region, regarding (1) logistic measures, such as percentage over- and undertriage and patient distribution, (2) patient measures, such as the percentage of IVT/EVT within 4.5 and 6 hours respectively, complications and the distribution of diagnoses such as intracerebral hemorrhage and the corresponding triage advice, and (3) user measures, such as the number of monthly users of the Stroke Triage App, and compliance amongst paramedics.*

For the secondary objective, we will use collected data from the STA cohort. This dataset contains data gathered in the ambulance that is entered into the STA, data which are automatically generated by the STA, and data from the hospital electronic files. After the follow-up of the final patients, the last records of the database will be cleaned and checked for completeness within one month. Upon completion, the database will be locked.

Missing data

We will report proportions of missing values for all collected variables. Because the secondary objective only involves descriptive statistics, imputation of missing values is not applicable. Only the crude, non-imputed data will be presented.

Data analysis

The study protocol specified that the STA should be used for all adult patients (≥18 years) with a suspected stroke (based on a positive FAST-test (≥1 point)) with onset of symptoms within the last 24 hours. For these results, data from all adult patients for whom the app was utilized will be included, following an intention-to-treat analysis mimicking clinical practice. Patients for whom the STA was entered after arrival at the emergency department, were excluded from analysis, as this could distort the results of the primary outcome. Patients without a suspected stroke for which the STA was used, were also excluded.

1. Logistic metrics
    - Patient distribution, percentage under- and overtriage, correctly bypassed patients

2. Patient metrics
    - Percentage of EVT, IVT, and percentage of treatments within conventional treatment windows (IVT: <4,5 hours, EVT: <6 hours), door to groin, door to needle times
3. User metrics
    - Monthly users of the Stroke Triage App, number of monthly triages, user compliance

In this objective, data regarding geographical distribution of patients, treatment characteristics (such as IVT/EVT within time windows) and triage characteristics (percentage under- and overtriage, correctly bypassed patients) will be analyzed. An overview regarding patient characteristics will be developed with e.g. age, NIHSS score, LVO yes/no, tension, age, sex, medical history and medication use. In addition, secondary outcomes such as onset to groin, onset to needle times, percentage of treatment within conventional treatment windows, and mRS score at 90 days will be evaluated. Time to treatment for both IVT and EVT will be analyzed and presented, as well as the number of correctly triaged patients. In addition, organizational outcomes such as workflow times will be analyzed. For user metrics, we will investigate the number of monthly STA users, numbers of monthly triages, and user compliance.
