# Supplementary material for: Prehospital triage of patients with suspected acute stroke (PRESTO-2): protocol for a controlled before-and-after intervention trial
Source: Eur Stroke J. 2026 Sep 7;11(9):aakag106. doi: 10.1093/esj/aakag106 (PMC13547856; doi:10.1093/esj/aakag106)
Supplement: Supplementary file 1 [file supplementary_material_aakag106.zip › Supplemental_material_caption.docx]

**Supplemental material**

**Document 1.** Stroke Triage App – Intended Purpose and Classification

**Document 2.** Statistical Analysis Plan
